# Association between the Survey-based Women's Empowerment (SWPER) index and barriers to healthcare in sub-Saharan Africa

**DOI:** 10.1093/inthealth/ihaf023

**Published:** 2025-05-20

**Authors:** Richard Gyan Aboagye, Mainprice Akuoko Essuman, Tarif Salihu, Abdul-Aziz Seidu, John Elvis Hagan, Frank Baiden, Bright Opoku Ahinkorah

**Affiliations:** School of Population Health, University of New South Wales, Sydney, NSW 2052, Australia; Department of Family and Community Health, Fred N. Binka School of Public Health, University of Health and Allied Sciences, Hohoe, Ghana; Department of Medical Laboratory Science, School of Allied Health Sciences, College of Health and Allied Sciences, University of Cape Coast, Cape Coast, Ghana; Department of Population and Health, University of Cape Coast, Cape Coast, Ghana; Public Health and Tropical Medicine, James Cook University, Cook University, QLD 4811, Australia; Neurocognition and Action-Biomechanics-Research Group, Faculty of Psychology and Sport Sciences, Bielefeld University, Bielefeld, Germany; Department of Health, Physical Education, and Recreation, University of Cape Coast, Cape Coast, Ghana; Department of Epidemiology and Biostatistics, Fred N. Binka School of Public Health, University of Health and Allied Sciences, Hohoe, Ghana; Faculty of Health and Medical Sciences, The University of Adelaide, Adelaide, Australia

**Keywords:** barriers, healthcare access, SWPER, women's empowerment

## Abstract

**Background:**

Women's health is an essential component of the Sustainable Development Goals. We examined how women's empowerment influences barriers to healthcare access in sub-Saharan Africa.

**Methods:**

The study included a weighted sample of 188 572 women's data from the Demographic and Health Surveys of 21 countries. A multilevel binary logistic regression analysis was used to examine the association between women's empowerment and barriers to accessing healthcare.

**Results:**

Women in the medium and high categories of attitude towards violence, social independence and autonomy were less likely to experience problems in getting permission to go for treatment. Women who had high attitudes towards violence were less likely to experience problems in getting money for treatment. Women with high social independence were less likely to face problems getting money for treatment. Women with high scores for attitudes towards violence, social independence and decision-making had the lowest likelihood of experiencing difficulty with distance to a health facility. Similarly, those in the high categories for attitude towards violence, social independence and decision-making had the lowest odds of experiencing problems not wanting to go alone.

**Conclusions:**

Women's empowerment decreases the barriers to accessing healthcare in sub-Saharan Africa. Designing healthcare intervention programs for women should consider the contribution that women make to household decision-making, social independence and attitudes towards violence.

## Introduction

Women's health is an important issue with a lot of emphasis placed on the need to eliminate all barriers. Sustainable Development Goal 3 (SDG) targets 3.7 and 3.8 aim to ensure universal access to essential healthcare services and integrate reproductive healthcare into national policies and programs, and to achieve universal health coverage by 2030, respectively.^1^ The Global Strategy for Women's, Children's and Adolescents’ Health^2^ and the World Health Organization Global Action Plan^3^ have been introduced to improve women's health. By enhancing women's access to and use of health services, health concerns such as pregnancy and birth, prenatal and neonatal mortality, maternal morbidity and death and vertical transmission of infectious diseases affecting women could be addressed.^4,5^ Although some efforts have been made to improve women's health globally, many efforts still need to be made. Many women in low- and middle-income countries (LMICs) experience challenges in accessing healthcare, which has led to worse health outcomes.^6,7^ A total of 6 in 10 women in sub-Saharan Africa (SSA) report facing barriers to accessing healthcare.^8^

Access to healthcare is usually measured through health service utilization. Women in SSA have reported challenges in seeking medical care across different Demographic and Health Surveys (DHS).^9,10^ Barriers to accessing healthcare during pregnancy^11,12^ or for their sick children^13^ have also been reported among these women. The barriers come in many forms, which may not be limited to financial (possession of or perceived ability to obtain financial resources), geographic (distance and means of transportation), as well as sociocultural (e.g. worries about getting permission and going alone).^14–17^ Studies that have evaluated the factors affecting healthcare access among women usually put these issues together without investigating how these factors are independently influenced.^8^ It is important to pay close attention to these barriers impeding women's healthcare and holistically address them.

Although not widely explored, women's empowerment may influence the use of health services.^18^ Empowerment is frequently described as both a process and a result that gives people control over their lives and decisions.^19^ Empowerment can be defined as ‘the process of enhancing an individual's or group's capacity to make purposeful choices and to transform those choices into desired actions and outcomes’.^20^ Empowerment of women involves making them use their resources, opportunities and agency to make deliberate decisions and act in ways that change their circumstances for the better. Besides the social impact of empowering women, it also influences the health of women and their families. In male-dominated communities like SSA, men frequently make choices regarding their wives and children's health, including the family's use of medical facilities.^21,22^ However, empowering women may influence how the household utilizes healthcare. Women's empowerment could influence the use of contraception, reduced fertility and longer birth intervals.^23^ Any community's health and wealth are directly linked to that of its women and girls, but only by empowering them to achieve equality.^24^

One unique instrument for defining African women's empowerment is the Survey-based Women's Empowerment (SWPER) index.^25,26^ The SWPER index measures three domains of women's empowerment—social independence, decision-making and attitude towards violence. It also employs individual-level data, allowing for analysing connections between empowerment and different health treatments and outcomes. Although all three dimensions captured by the SWPER index have the potential to influence women's access to healthcare, no study has been conducted in SSA examining its association with barriers to accessing healthcare using a nationally representative survey dataset.

Literature on the association between women's empowerment and barriers to healthcare access is woefully inadequate. Understanding how factors such as ‘obtaining permission for treatment’, ‘getting money for treatment’, ‘distance to a health facility’ and ‘not wanting to go alone’ serve as common barriers to women's access to healthcare^27^ would help develop appropriate interventions. Using the novel, multidimensional and validated SWPER index, the study examined how women's empowerment influences the most common barriers to accessing healthcare in SSA. The findings of this study will provide useful information for designing effective gender-based policies and programs in healthcare.

## Methods

### Data source and study design

This study was conducted among 188 572 women of reproductive age (15–49 y) across 21 countries in SSA using recent data from the DHS from 2015 to 2021. This sample consisted of women who were married or cohabiting at the time of the survey. The DHS is a nationwide survey conducted in >90 LMICs,^28^ using a cross-sectional design, with respondents selected using a two-stage cluster sampling technique.^29^ Data were pooled from the women's files in the 21 countries. The list of the selected countries, their corresponding survey years and the respective sample proportions are presented in Table 1. Only countries that had observations on the variables of interest—in sexual unions: married or cohabiting, as well as all the outcome and explanatory variables—were included in the study (Table 1). In the DHS, pretested and validated structured questionnaires were used to collect data from the respondents.^28,29^ We followed the Strengthening the Reporting of Observational Studies in Epidemiology guidelines to develop this article.^30^

**Table 1.** Description of study sample per country

Country	Year of survey	Weighted sample	Weighted percentage
Angola	2015–16	8137	4.31
Benin	2017–18	9015	4.78
Burundi	2016–17	10 016	5.31
Cameroon	2018	8673	4.60
Ethiopia	2016	9246	4.90
Gambia	2019–20	6145	3.26
Guinea	2018	6096	3.23
Liberia	2019–20	4182	2.22
Madagascar	2021	10 772	5.71
Mali	2018	6122	3.25
Mauritania	2019–2021	8862	4.70
Malawi	2015–16	14 075	7.46
Nigeria	2018	23 787	12.61
Rwanda	2019–20	8427	4.47
Sierra Leone	2019	8697	4.61
Chad	2014–15	9667	5.13
Tanzania	2015–16	7556	4.01
Uganda	2016	10 311	5.47
South Africa	2016	5218	2.77
Zambia	2018	7811	4.14
Zimbabwe	2015	5757	3.05
All countries	2015–2021	188 572	100.00

### Variables

The study used barriers to accessing healthcare as outcome variables. In the DHS, women were asked to report serious problems they encountered in accessing healthcare according to the type of problem. The women mentioned four problems, which were getting medical help for self: getting permission to go (to a health facility), getting money needed for treatment, distance to a health facility and not wanting go (to health facilities) alone. The response options for each of these were ‘big problem’ and ‘not a big problem’. For our final analysis, we recoded the response option into 0 (not a big problem) and 1 (big problem). Previous studies have utilized these variables individually or as composites to measure barriers to accessing healthcare.^8,9,11,23,31^

Women's empowerment was the key explanatory variable in this study, and was determined using the SWPER index. The SWPER index was developed, tested and validated for use in LMICs.^25^ The SWPER index has been applied to several health and social issues encompassing maternal and child health and reproductive health, among others.^26,32^ However, its influence on women's accessibility to healthcare has not been explored in the sub-Saharan African subregion, hence the selection of the SWPER index as a key explanatory variable. Fourteen variables in the DHS were used to develop the SWPER index, which was categorized into three dimensions of women’s empowerment: attitude towards violence, social autonomy and decision-making.^25^ These categories of the SWPER index were utilized in the study to examine its effect on barriers to accessing healthcare. However, the subcategorization and coding of the SWPER index dimensions align with existing literature.^32,33^ Hence, each dimension of the SWPER index was categorized as ‘low’, ‘medium’ and ‘high’. For attitude towards violence, the high category represents high disagreement or rejection of an attitude towards violence (positive), with the low category emphasizing high acceptance of violence (negative).

Eight variables were controlled for in the study. These variables were selected based on their influence on barriers to accessing healthcare from the literature^8,9,11,23,31^ and their availability in the DHS dataset. The covariates were classified into individual- and contextual-level variables based on previous studies.^8,9,11,23,31^ The individual-level variables consisted of parity, health insurance coverage, exposure to radio and exposure to television. Household wealth index, sex of the household head, place of residence and geographic subregions were the contextual-level variables.

### Statistical analyses

Stata version 17.0 (StataCorp, College Station, TX, USA) was used for all the analyses. The proportion of women who encountered barriers in accessing healthcare was presented using percentages and on spatial maps. Using cross-tabulation analysis, we examined the distribution of barriers to accessing healthcare across the dimensions of the SWPER index and the covariates. The Pearson χ^2^ test of independence was used to determine the variables significantly associated with the barriers to accessing healthcare. A follow-up multilevel binary logistic regression analysis was used to examine the association between SWPER index and barriers to access healthcare using five models. Model O was an empty model with no key explanatory variable or covariate. Model I contained the key explanatory variables. Model I and the individual level covariates were placed in model II. Model III contained model I and the contextual level covariates. Model IV was the complete model and it was fitted to contain model I and all the covariates. The regression results were presented using adjusted odds ratios (AORs) with 95% confidence intervals (CIs). We used the Akaike information criterion (AIC) and log-likelihood values to assess the fitness of the models. The model with the lowest AIC and highest log-likelihood value was chosen as the best-fitted model and its results were interpreted and discussed. Model IV was chosen as the best-fitted model. Statistical significance at the χ^2^ and regression level was set at p<0.05. All the analyses were weighted according to DHS guidelines.^28^

### Multilevel modelling

The equations for the multilevel binary logistic regression models can be described as follows:

- *Y_ij_* denotes each outcome variable: getting medical help for self, getting permission to go, getting the money needed for treatment, distance to a health facility and not wanting to go alone for the individual *i* in cluster (or level 2 unit) *j.*
- *AV_ij_, SI_ij_* and *DM_ij_* indicate the three dimensions of the SWPER index (attitude towards violence, social independence and decision-making) for individual *i* in cluster *j.*
- *Covariates_ij_* is a vector of covariates (parity, health insurance coverage, exposure to radio, exposure to television, sex of the household head, household wealth index, place of residence and geographic subregion for individual *i* in cluster *j*.

\begin{eqnarray*}
\mathit{logit}\left( {\frac{{P\left( {{Y_{ij}} = 1} \right)}}{{1 - P\left( {{Y_{ij}} = 1} \right)}}} \right) = \beta {o_j} + {u_j} + \,\,{e_{ij}}
\end{eqnarray*}

for the model with no explanatory variable.


\begin{eqnarray*}
\mathit{logit}\left( {\frac{{P\left( {{Y_{ij}} = 1} \right)}}{{1 - P\left( {{Y_{ij}} = 1} \right)}}} \right) &=& \beta 0 + {\beta _{AV}}A{V_{ij}} + {\beta _{SI}}S{I_{ij}}\\
&& +\, {\beta _{DM}}D{M_{ij}} + {u_j} + \,\,{e_{ij}}
\end{eqnarray*}

for the model with the dimensions of the SWPER index.


\begin{eqnarray*}
\mathit{logit}\left( {\frac{{P\left( {{Y_{ij}} = 1} \right)}}{{1 - P\left( {{Y_{ij}} = 1} \right)}}} \right) &=& \beta 0 + {\beta _{AV}}A{V_{ij}} + {\beta _{SI}}S{I_{ij}} + {\beta _{DM}}D{M_{ij}}\\
&& + {\beta _{COV}}\mathit{Covariate}{s_{ij}} + {u_j} + \,\,{e_{ij}}
\end{eqnarray*}

for the model with the dimensions of the SWPER index and individual-level covariates (parity, health insurance coverage, exposure to radio and exposure to television).


\begin{eqnarray*}
\mathit{logit}\left( {\frac{{P\left( {{Y_{ij}} = 1} \right)}}{{1 - P\left( {{Y_{ij}} = 1} \right)}}} \right) &=& \beta 0 + {\beta _{AV}}A{V_{ij}} + {\beta _{SI}}S{I_{ij}} + {\beta _{DM}}D{M_{ij}}\\
&& + {\beta _{COV2}}\mathit{Covariate}{s_{ij2}} + {u_j} + {e_{ij}}
\end{eqnarray*}

for the model with the dimensions of the SWPER index and the contextual-level variables (sex of the household head, household wealth index, place of residence, and geographic subregion).


\begin{eqnarray*}
&&\mathit{logit}\left( {\frac{{P\left( {{Y_{ij}} = 1} \right)}}{{1 - P\left( {{Y_{ij}} = 1} \right)}}} \right) = \beta 0 + {\beta _{AV}}A{V_{ij}} + {\beta _{SI}}S{I_{ij}} + {\beta _{DM}}D{M_{ij}} \\
&&\qquad + {\beta _{COV}}\mathit{Covariate}{s_{ij}} + {\beta _{COV2}}\mathit{Covariate}{s_{ij2}} + {u_j} + \,\,{e_{ij}}
\end{eqnarray*}

for the model with dimensions of the SWPER index and all the covariates.

## Results

### Proportion of indicators of barriers to access healthcare per country

**Figure 1.**
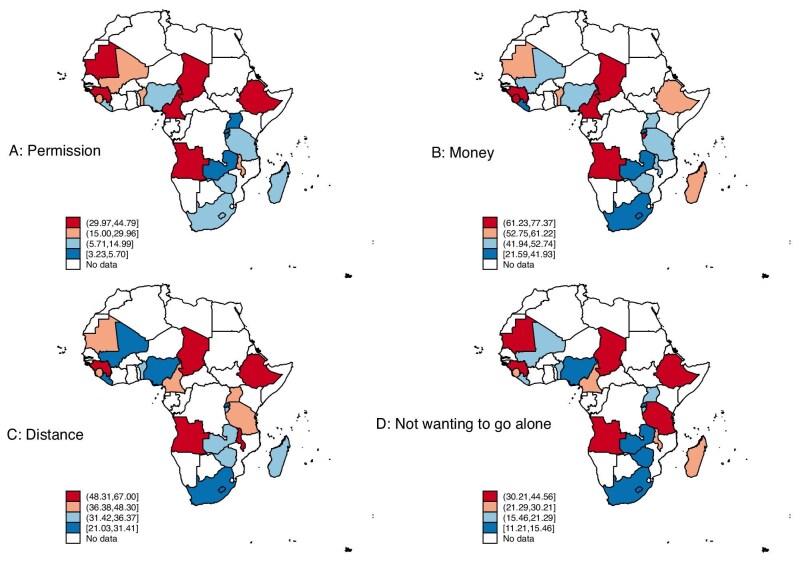
Proportion of barriers to healthcare per country. The figures are choropleth maps showing the proportion of women in the surveyed countries who indicated (A) getting permission to go (to a health facility), (B) getting money needed for treatment, (C) distance to a health facility and (D) not wanting to go (to health facilities) alone as barriers to accessing healthcare. The values within square brackets in the colour legends indicate the proportion (%) of women who the stated reason as barrier for accessing healthcare.

Figure 1 presents the proportion of women facing the stated barriers to healthcare per country. From Figure 1A, the hotspot countries where women had problems getting permission to go for treatment were Chad, Cameroon, Ethiopia, Mauritania and Angola. For problems in getting money for treatment, the hotspot countries were Chad, Sierra Leone, Burundi, Cameroon, Angola and Guinea (Figure 1B). Chad, Malawi, Ethiopia, Angola, Guinea and Sierra Leone were the hotspot countries where the distance to a health facility was a barrier to healthcare access (Figure 1C). From Figure 1D, not wanting to go to health facilities alone was a predominant barrier to healthcare access in Chad, Ethiopia, Guinea, Tanzania, Mauritania and Angola. Detailed results on the distribution of each barrier per country can be found in Supplementary Table S1.

### Distribution of barriers to access healthcare across the explanatory variables

Table 2 shows the distribution of barriers to accessing healthcare across the dimensions of the SWPER index. Across all the dimensions of the SWPER index, the proportion of women experiencing barriers to accessing healthcare in all four indicators decreases as the level of each dimension increases. Across the four barriers to accessing healthcare, the highest proportion of barriers was recorded among women in the low category in each dimension of the SWPER index. Also, all three dimensions of the SWPER index were significantly associated with the barriers to accessing healthcare at p<0.001. Except for the sex of the household head, which was only associated with not wanting to go to health facilities alone, the remaining covariates showed a statistically significant association with the four barriers to access healthcare at p<0.05.

**Table 2.** Distribution of barriers to access healthcare across the explanatory variables

Variable	Weighted n (%)	Getting permission to go	p-Value	Getting money needed for treatment	p-Value	Distance to a health facility	p-Value	Not wanting to go alone	p-Value
Attitude to violence			<0.001		<0.001		<0.001		<0.001
Low	49 882 (26.4)	23.7		57.0		45.3		29.0	
Medium	33 294 (17.7)	17.7		52.7		40.5		24.0	
High	105 396 (55.9)	16.3		48.4		36.7		21.3	
Social independence (autonomy)			<0.001		<0.001		<0.001		<0.001
Low	59 216 (31.4)	24.3		59.5		46.9		28.9	
Medium	65 053 (34.5)	18.3		54.1		42.3		24.7	
High	64 303 (34.1)	13.6		41.2		30.2		18.3	
Decision-making			<0.001		<0.001		<0.001		<0.001
Low	39 264 (20.8)	27.0		56.9		44.4		29.0	
Medium	89 162 (47.3)	18.1		52.7		41.1		24.2	
High	60 146 (31.9)	13.6		46.0		34.4		19.9	
Parity			<0.001		<0.001		<0.001		<0.001
Nulliparity	12 173 (6.5)	19.8		43.9		37.1		26.5	
Primiparity	27 611 (14.6)	17.4		45.6		36.0		22.8	
Multiparity	85 107 (45.1)	17.4		49.3		37.4		22.3	
Grand parity	63 681 (33.8)	20.2		58.2		44.7		25.8	
Covered by health insurance			<0.001		<0.001		<0.001		<0.001
No	171 453 (90.9)	19.7		53.4		41.5		25.0	
Yes	17 119 (9.1)	6.2		31.8		21.3		12.4	
Listens to radio			<0.001		<0.001		<0.001		<0.001
No	85 372 (45.3)	24.1		59.6		46.8		28.9	
Yes	103 200 (54.7)	13.8		44.7		33.7		19.6	
Watches television			<0.001		<0.001		<0.001		<0.001
No	117 531 (62.3)	21.2		59.5		47.9		28.6	
Yes	71 041 (37.7)	14.0		38.0		26.0		15.9	
Wealth index			<0.001		<0.001		<0.001		<0.001
Poorest	37 291 (19.8)	23.4		66.1		55.4		32.9	
Poorer	38 528 (20.4)	22.0		60.6		48.4		28.6	
Middle	37 492 (19.9)	19.2		53.9		42.0		24.4	
Richer	37 526 (19.9)	16.4		45.8		32.5		19.8	
Richest	37 734 (20.0)	11.5		30.7		19.9		13.5	
Sex of household head			0.907		0.351		0.157		0.021
Male	160 320 (85.0)	18.5		51.5		39.8		24.0	
Female	28 252 (15.0)	18.5		51.0		39.0		23.0	
Place of residence			<0.001		<0.001		<0.001		<0.001
Urban	62 886 (33.3)	14.4		39.0		23.0		14.4	
Rural	125 686 (66.6)	20.5		57.7		48.0		28.6	
Geographic subregion			<0.001		<0.001		<0.001		<0.001
Central Africa	26 477 (14.0)	37.2		70.1		54.4		34.4	
Southern Africa	18 785 (10.0)	6.5		29.4		30.4		12.7	
Eastern Africa	70 403 (37.3)	13.6		51.9		41.8		26.5	
Western Africa	72 907 (38.7)	19.6		49.9		34.6		20.4	

*The p-values are generated from the χ^2^ test.

### Association between the SWPER index and problem accessing healthcare: getting permission to go for treatment

Table 3 presents the association between the SWPER index and problems accessing healthcare in terms of getting permission to go for treatment. The results showed that the odds of women facing problems in getting permission to go for treatment decreases in all the dimensions of the SWPER index as each dimension's category increases from low to high. The odds of experiencing problems in getting permission to go for treatment decreased as attitude towards violence increased from a negative connotation to positive, with the lowest odds among women in the high attitude towards violence (positive) (AOR 0.78 [95% CI 0.73 to 0.83]). Compared with women in the low category for social independence and autonomy, those in the high social independence (AOR 0.89 [95% CI 0.85 to 0.95]) and high decision-making (AOR 0.66 [95% CI 0.62 to 0.70]) categories had the lowest odds of experiencing a problem in getting permission to go for treatment.

**Table 3.** Association between the SWPER index and problem accessing healthcare: getting permission to go for treatment

Variables	Model O	Model I, AOR (95% CI)	Model II, AOR (95% CI)	Model III, AOR (95% CI)	Model IV, AOR (95% CI)
Fixed effects model					
Attitude towards violence					
Low (reference)		1.00	1.00	1.00	1.00
Medium		0.77*** (0.72 to 0.82)	0.78*** (0.74 to 0.83)	0.81*** (0.76 to 0.87)	0.81*** (0.76 to 0.86)
High		0.75*** (0.70 to 0.80)	0.75*** (0.71 to 0.80)	0.79*** (0.74 to 0.84)	0.78*** (0.73 to 0.83)
Social independence (autonomy)					
Low (reference)		1.00	1.00	1.00	1.00
Medium		0.74*** (0.71 to 0.77)	0.79*** (0.76 to 0.82)	0.88*** (0.84 to 0.92)	0.90*** (0.86 to 0.94)
High		0.58*** (0.55 to 0.62)	0.73*** (0.69 to 0.77)	0.82*** (0.77 to 0.86)	0.89*** (0.85 to 0.95)
Decision-making					
Low (reference)		1.00	1.00	1.00	1.00
Medium		0.63*** (0.60 to 0.67)	0.68*** (0.64 to 0.72)	0.74*** (0.70 to 0.79)	0.77*** (0.72 to 0.82)
High		0.50*** (0.46 to 0.53)	0.56*** (0.52 to 0.59)	0.62*** (0.58 to 0.67)	0.66*** (0.62 to 0.70)
Random effects model					
PSU variance (95% CI)	0.73 (0.61 to 0.86)	0.66 (0.55 to 0.79)	0.66 (0.55 to 0.79)	0.63 (0.53 to 0.75)	0.64 (0.53 to 0.76)
ICC	0.18	0.17	0.17	0.16	0.16
Wald χ^2^	Reference	1105.69***	1628.87***	1791.40***	2165***
Model fitness					
Log-likelihood	−154 833.04	−150 999.93	−148 260	−144 049.72	−142 659.77
AIC	309 670.1	302 015.9	296 548	288 133.4	285 365.5
N	188 572	188 572	188 572	188 572	188 572
Number of clusters	1399	1399	1399	1399	1399

*p<0.05, **p<0.01, ***p<0.001.

PSU: primary sampling unit; ICC: intraclass correlation coefficient.

### Association between the SWPER index and problem accessing healthcare: getting money for treatment

Table 4 displays the association between the SWPER index and problems accessing healthcare regarding getting money for treatment. Women who belonged to the high attitude towards violence category were less likely to experience problems in getting money for treatment compared with those in the low category (AOR 0.94 [95% CI 0.90 to 0.98]). Relative to women in the low social independence category, those in the high category were less likely to face problems in getting money for treatment (AOR 0.95 [95% CI 0.91 to 0.98]).

**Table 4.** Association between the SWPER index and problem accessing healthcare: getting money for treatment

Variable	Model O	Model I, AOR (95% CI)	Model II, AOR (95% CI)	Model III, AOR (95% CI)	Model IV, AOR (95% CI)
Fixed effects model					
Attitude towards violence					
Low (reference)		1.00	1.00	1.00	1.00
Medium		0.90*** (0.86 to 0.94)	0.93** (0.89 to 0.98)	0.97^NS^ (0.93 to 1.02)	0.98^NS^ (0.93 to 1.02)
High		0.81*** (0.78 to 0.85)	0.86*** (0.82 to 0.90)	0.94** (0.90 to 0.98)	0.94** (0.90 to 0.98)
Social independence (autonomy)					
Low (reference)		1.00	1.00	1.00	1.00
Medium		0.83*** (0.80 to 0.85)	0.92*** (0.89 to 0.95)	0.96* (0.93 to 0.99)	1.01^NS^ (0.98 to 1.05)
High		0.53*** (0.51 to 0.55)	0.76*** (0.73 to 0.78)	0.80*** (0.77 to 0.83)	0.95*** (0.91 to 0.98)
Decision-making					
Low (reference)		1.00	1.00	1.00	1.00
Medium		0.92*** (0.88 to 0.96)	0.96^NS^ (0.92 to 1.00)	1.00^NS^ (0.95 to 1.05)	1.02^NS^ (0.97 to 1.06)
High		0.77*** (0.73 to 0.81)	0.87*** (0.83 to 0.91)	0.96^NS^ (0.91 to 1.01)	0.98^NS^ (0.94 to 1.04)
Random effects model					
PSU variance (95% CI)	0.53 (0.44 to 0.63)	0.46 (0.38 to 0.55)	0.42 (0.35 to 0.51)	0.41 (0.34 to 0.50)	0.41 (0.34 to 0.50)
ICC	0.14	0.12	0.11	0.11	0.11
Wald χ^2^	Reference	1411.73***	2940.09***	3918.19***	4384.79***
Model fitness					
Log-likelihood	−225 876.65	−221 853.87	−215 307.84	−209 170.35	−206 848.52
AIC	451 757.3	443 723.7	430 643.7	418 374.7	413 743
N	188 572	188 572	188 572	188 572	188 572
Number of clusters	1399	1399	1399	1399	1399

*p<0.05, **p<0.01, ***p<0.001.

PSU: primary sampling unit; ICC: intraclass correlation coefficient; NS: non-significant.

### Association between the SWPER index and problems accessing healthcare: distance to a health facility

Table 5 presents the association between the SWPER index and problems accessing healthcare in terms of distance to a health facility. Women belonging to the high categories in all the dimensions of the SWPER index were less likely to experience difficulty with distance to a health facility relative to those in the low category. Thus, women with high attitude towards violence (AOR 0.94 [95% CI 0.89 to 0.99]), high social independence (AOR 0.90 [95% CI 0.87 to 0.94]) and those with high decision-making (AOR 0.89 [95% CI 0.84 to 0.94]) had the lowest likelihood of experiencing difficulty with distance to a health facility.

**Table 5.** Association between the SWPER index and problem accessing healthcare: distance to a health facility

Variable	Model O	Model I, AOR (95% CI)	Model II, AOR (95% CI)	Model III, AOR (95% CI)	Model IV, AOR (95% CI)
Fixed effects model					
Attitude towards violence					
Low (reference)		1.00	1.00	1.00	1.00
Medium		0.88*** (0.84 to 0.92)	0.91*** (0.87 to 0.95)	0.95* (0.90 to 0.99)	0.94* (0.90 to 0.99)
High		0.80*** (0.76 to 0.85)	0.86*** (0.81 to 0.90)	0.95* (0.90 to 1.00)	0.94* (0.89 to 0.99)
Social independence (autonomy)					
Low (reference)		1.00	1.00	1.00	1.00
Medium		0.861*** (0.83 to 0.89)	0.94** (0.91 to 0.97)	0.95** (0.91 to 0.98)	0.98^NS^ (0.94 to 1.01)
High		0.55*** (0.53 to 0.58)	0.77*** (0.74 to 0.80)	0.79*** (0.76 to 0.82)	0.90*** (0.87 to 0.94)
Decision-making					
Low (reference)		1.00	1.00	1.00	1.00
Medium		0.95^NS^ (0.91 to 1.00)	1.00^NS^ (0.95 to 1.05)	0.96^NS^ (0.91 to 1.01)	0.98^NS^ (0.93 to 1.03)
High		0.79*** (0.75 to 0.84)	0.90*** (0.85 to 0.95)	0.86*** (0.82 to 0.91)	0.89*** (0.84 to 0.94)
Random effects model					
PSU variance (95% CI)	1.10 (0.93 to 1.32)	0.92 (0.76 to 1.12)	0.78 (0.64 to 0.96)	0.73 (0.59 to 0.90)	0.71 (0.57 to 0.87)
ICC	0.25	0.22	0.19	0.18	0.18
Wald χ^2^	Reference	1039.26***	2390.14***	3190.17***	3599.79***
Model fitness					
Log-likelihood	−217 164.1	−213 724.57	−207 288.75	−201 083.16	−199 223.81
AIC	434 332.2	427 465.1	414 605.5	402 200.3	398 493.6
N	188 572	188 572	188 572	188 572	188 572
Number of clusters	1399	1399	1399	1399	1399

*p<0.05, **p<0.01, ***p<0.001.

PSU: primary sampling unit; ICC: intraclass correlation coefficient; NS: non-significant.

### Association between the SWPER index and problem accessing healthcare: not wanting to go alone

Results from Table 6 show that the likelihood of women experiencing problems not wanting to go alone decreases in all the dimensions of the SWPER index as the dimension's categories move from low to high. Compared with women in the low categories for each dimension, those in the high categories for attitude towards violence (AOR 0.83 [95% CI 0.79 to 0.88]), social independence (AOR 0.90 [95% CI 0.86 to 0.94]), and decision-making (AOR 0.78 [95% CI 0.73 to 0.83]) had the lowest odds of experiencing a problem not wanting to go alone.

**Table 6.** Association between the SWPER index and problem accessing healthcare: not wanting to go alone

Variable	Model O	Model I, AOR (95% CI)	Model II, AOR (95% CI)	Model III, AOR (95% CI)	Model IV, AOR (95% CI)
Fixed effects model					
Attitude towards violence					
Low (reference)		1.00	1.00	1.00	1.00
Medium		0.83*** (0.79 to 0.87)	0.85*** (0.81 to 0.89)	0.86*** (0.82 to 0.91)	0.86*** (0.81 to 0.90)
High		0.75*** (0.71 to 0.79)	0.78*** (0.74 to 0.82)	0.84*** (0.80 to 0.89)	0.83*** (0.79 to 0.88)
Social independence (autonomy)					
Low (reference)		1.00	1.00	1.00	1.00
Medium		0.85*** (0.82 to 0.88)	0.89*** (0.86 to 0.93)	0.90*** (0.87 to 0.94)	0.92*** (0.88 to 0.95)
High		0.63*** (0.61 to 0.66)	0.80*** (0.76 to 0.83)	0.83*** (0.79 to 0.86)	0.90*** (0.86 to 0.94)
Decision-making					
Low (reference)		1.00	1.00	1.00	1.00
Medium		0.84*** (0.79 to 0.88)	0.88*** (0.83 to 0.93)	0.82*** (0.77 to 0.86)	0.84*** (0.80 to 0.89)
High		0.71*** (0.67 to 0.75)	0.79*** (0.75 to 0.84)	0.74*** (0.70 to 0.79)	0.78*** (0.73 to 0.83)
Random effects model					
PSU variance (95% CI)	0.74 (0.62 to 0.90)	0.63 (0.52 to 0.76)	0.58 (0.48 to 0.71)	0.54 (0.44 to 0.66)	0.54 (0.44 to 0.66)
ICC	0.18	0.16	0.15	0.14	0.14
Wald χ^2^	Reference	766.72***	1373.31***	1847.87***	2036.74***
Model fitness					
Log-likelihood	−178 681.45	−176 348.16	−173 332.01	−170 240.74	−168 977.65
AIC	357 366.9	352 712.3	346 692	340 515.5	338 001.3
N	188 572	188 572	188 572	188 572	188 572
Number of clusters	1399	1399	1399	1399	1399

*p<0.05, **p<0.01, ***p<0.001.

PSU: primary sampling unit; ICC: intraclass correlation coefficient.

## Discussion

This study examined the association between dimensions of the SWPER index (attitude towards violence, autonomy, decision-making) and barriers to healthcare access among women in SSA. The findings indicated that barriers to healthcare accessibility are predominant among women in SSA, with getting money for treatment, distance to a health facility, getting permission to go for treatment and not wanting to go alone as the main barriers. The hotspot countries where women had problems getting permission to go for treatment were Chad, Cameroon, Ethiopia, Mauritania and Angola. For problems in getting money for treatment, the hotspot countries were Chad, Sierra Leone, Burundi, Angola and Guinea. In terms of distance to a health facility, Chad, Malawi, Ethiopia, Angola, Guinea and Sierra Leone were the hotspot countries. As another main barrier to healthcare access, not wanting to go alone was also found to be predominant in Chad, Ethiopia, Guinea, Tanzania, Mauritania and Angola. It is worth noting that women in Chad showed the highest proportion of barriers to healthcare access in SSA in terms of getting permission to go for treatment, getting money for treatment, distance to a health facility and not wanting to go alone. The high percentages of barriers to healthcare among Chadian women could be attributed to socio-economic factors such as a lack of funds for transportation and fees, inadequate healthcare facilities, poor road networks and a preference for traditional services.^8,34^

Similar to previous studies in SSA,^35–37^ the degree to which women use healthcare services can be influenced by markers of women's empowerment. In this study, barriers were examined by asking participants if they had difficulty receiving medical care because they needed permission, money, distance to a health facility or they did not want to travel alone. These indicators had a detrimental influence on SSA residents’ ability to receive healthcare in various situations.^23,38^ In a qualitative study conducted in West African countries, ineffective health decision choices and the high cost of healthcare were the two main obstacles that prevented women from receiving the care they needed.^23,39^ Women who scored high on empowerment faced fewer obstacles, supporting earlier research that women's empowerment had a major effect on access to healthcare.^18,23^

Women in SSA with positive attitudes towards violence were less likely than women with low attitudes towards violence to experience difficulties in accessing healthcare as compared with those with a low attitude towards violence.^40^ Women's attitudes towards violence have a great impact on their health-seeking behaviors.^40^ Women with a positive attitude against violence were less likely to have access problems. Women have the prerogative and empowerment (decision-making power, financial autonomy) to determine a safer health option or to decide whether to seek healthcare for themselves and their children.

Compared with women who did not have strong social independence (autonomy), those who had strong social independence had a lower likelihood of having trouble getting access to healthcare. A sign of a woman's control over her social life is her ability to pay a visit to friends and family whenever she wants. Women who experience a high degree of independence, including the ability to visit friends and family, are better equipped to interact frequently with the formal health system. Previous studies conducted in Nigeria^35,41^ found that women who report having the liberty to visit friends and family are significantly less likely to have problems obtaining healthcare services than those who did not report having this liberty. Notably, women with more autonomy make better use of healthcare resources for themselves and their children.^41,42^ Women's autonomy has been acknowledged as an empowerment indicator that could improve health outcomes for women.^41,42^ Women's access to resources, their ability to make decisions and addressing gender inequality in homes should be a priority when designing interventional programs to increase women's contact with health services in SSA countries.^41,42^

Other results showed that compared with women with poor decision-making capacity, those with greater decision-making capacity were less likely to encounter any issues when seeking medical care. Women who engaged in health decision-making were less likely to have problems seeking healthcare at a hospital. This outcome is consistent with results from several other surveys.^35,37,41^ Ameyaw and Dickson^43^ assert that women who have autonomy over their health decisions are less likely than their peers to face barriers to accessing healthcare. Sadly, women in many households in sub-Saharan African countries do not contribute considerably to decisions about their health, mainly because many families deeply ascribe to patriarchal norms.^35^ Usually, men make most decisions about the welfare of their wives and children. Decisions made jointly by a husband and wife greatly influence the likelihood that couples would use healthcare services.^44^ Therefore, it would be beneficial for countries in SSA to adopt intervention programs (e.g. cooperative learning and practical team-building exercises) that encourage or foster cooperative decisions at the household level to decrease power distances. Such programs could include educational materials informing men in SSA about the advantages of involving their spouses in decisions affecting their health and other family matters.

### Strengths and limitations

The key strength of this study is the use of nationally representative data to evaluate women's empowerment and its association with barriers to healthcare access among women in SSA. Despite this strength, the study's cross-sectional design precluded the inference of causality. The use of secondary data did not allow us to explore other factors, such as health worker–related factors, which can equally serve as barriers to healthcare for women. The self-reported and interview-based procedure used in collecting the DHS data is liable to social desirability bias. Because this study was based on secondary data analysis, the selected variables were only restricted to women's empowerment and barriers to healthcare access information already captured in the dataset. Other factors such as health insurance subscriptions and mass media serve as a proxy for empowerment and utilization of healthcare services in SSA. Future research could investigate the explicit roles of these factors in determining women's empowerment and healthcare access in SSA.

### Policy implications

The findings of this study provide useful information to guide public health policies in SSA and other LMICs. The achievement of SDG 3.1 may not be possible if strategies to improve women's empowerment are not enunciated and applied, per the results, which indicated that different dimensions of their empowerment significantly influence women's decision to utilize healthcare services.^41^ Policy efforts could be directed towards designing and implementing targeted healthcare interventions that specifically address the barriers identified in the SWPER index. The findings that lack of autonomy hinders women's access to healthcare call for interventions focused on empowering women through education and economic opportunities. Governments and organizations should consider implementing empowerment programs that aim to improve women's status in various dimensions, as highlighted by the SWPER index. By addressing factors such as education, financial independence and decision-making power, policymakers can work towards reducing barriers to healthcare. Policymakers could develop and implement health education campaigns that are focused on the specific challenges identified by the SWPER index. This might involve targeted messaging to raise awareness about women's rights to healthcare, reproductive health and the importance of seeking medical assistance. The association between the SWPER index and healthcare barriers may necessitate broader policy reforms. This could involve reviewing and modifying existing policies related to women's empowerment, healthcare accessibility and gender equality to ensure they align with the survey findings and address identified barriers. Policymakers should prioritize data-driven decision-making processes, utilizing the insights provided by the SWPER index to inform policy formulation and implementation. Regular monitoring and evaluation of these policies against the SWPER indicators will ensure that there are effective interventions that help reduce barriers to healthcare for women in SSA.

## Conclusions

The study showed that women's empowerment (attitude towards violence, social independence and decision-making) decreases barriers to healthcare among women in SSA. Designing healthcare intervention programs for women should be done taking into consideration the contribution that women make to household decision-making, their social independence and attitudes toward violence. Programs for intervention should be contextualized to reflect the unique circumstances of the women for whom they are intended. The implementation of national health insurance schemes and community health-based planning services could be useful programs to consider across countries in SSA. Various governments and developmental partners must pay particular attention to women's empowerment as a strategic intervention initiative to improve healthcare access across studied sub-Saharan African countries.

## Supplementary Material

ihaf023_Supplemental_File

## Data Availability

The datasets generated and/or analysed during the current study can be accessed from https://dhsprogram.com/data/available-datasets.cfm.
