# Supplementary material for: Association between the Survey-based Women's Empowerment (SWPER) index and barriers to healthcare in sub-Saharan Africa
Source: Int Health. 2025 May 20;17(5):734–44. doi: 10.1093/inthealth/ihaf023 (PMC12406787; doi:10.1093/inthealth/ihaf023)
Supplement: ihaf023_Supplemental_File [file ihaf023_supplemental_file.docx]

**Table S1: Proportion of barriers to access healthcare per country**

| **Country** | **Getting permission to go** | **Getting money needed for treatment** | **Distance to health facility** | **Not wanting to go alone** |
| --- | --- | --- | --- | --- |
| 1. Angola | 30.61 | 62.60 | 52.45 | 30.21 |
| 1. Benin | 23.10 | 53.24 | 33.02 | 18.42 |
| 1. Burundi | 5.36 | 62.69 | 33.0 | 16.27 |
| 1. Cameroon | 34.92 | 69.14 | 42.18 | 27.11 |
| 1. Ethiopia | 34.38 | 56.31 | 54.70 | 43.58 |
| 1. Gambia | 3.70 | 26.74 | 26.39 | 11.69 |
| 1. Guinea | 29.97 | 61.23 | 49.20 | 32.10 |
| 1. Liberia | 15.00 | 36.17 | 31.42 | 19.90 |
| 1. Madagascar | 14.81 | 55.44 | 35.24 | 30.18 |
| 1. Mali | 27.92 | 41.94 | 29.81 | 20.73 |
| 1. Mauritania | 30.83 | 54.46 | 42.65 | 30.26 |
| 1. Malawi | 16.48 | 52.75 | 56.41 | 29.71 |
| 1. Nigeria | 11.56 | 46.60 | 27.49 | 15.46 |
| 1. Rwanda | 3.23 | 40.57 | 22.14 | 12.09 |
| 1. Sierra Leone | 26.57 | 71.28 | 48.31 | 23.59 |
| 1. Chad | 44.79 | 77.37 | 67.00 | 44.56 |
| 1. Tanzania | 14.48 | 48.68 | 44.95 | 30.66 |
| 1. Uganda | 5.29 | 43.93 | 39.24 | 21.29 |
| 1. South Africa | 10.98 | 24.99 | 21.03 | 11.21 |
| 1. Zambia | 4.07 | 21.59 | 32.22 | 13.39 |
| 1. Zimbabwe | 5.72 | 44.10 | 36.38 | 13.13 |
| **All countries** | **18.50** | **51.42** | **39.65** | **23.85** |
